# Sitagliptin Alleviates Radiation-Induced Kidney and Testis Degeneration in Rats

**DOI:** 10.3390/biom15121702

**Published:** 2025-12-05

**Authors:** Huseyin Celik, Oztun Temelli, Onural Ozhan, Elif Taslidere, Feyzi Dogru

**Affiliations:** 1 Department of Urology, Faculty of Medicine, Inonu University, Malatya 44100, Türkiye; 2 Department of Radiation Oncology, Faculty of Medicine, Inonu University, Malatya 44100, Türkiye; oztun.temelli@inonu.edu.tr; 3 Department of Pharmacology, Faculty of Medicine, Inonu University, Malatya 44100, Türkiye; onural.ozhan@inonu.edu.tr; 4 Department of Histology and Embryology, Faculty of Medicine, Inonu University, Malatya 44100, Türkiye; elif.taslidere@inonu.edu.tr; 5 Department of Physiology, Faculty of Medicine, Turgut Ozal University, Malatya 44100, Türkiye; feyzi.dogru@ozal.edu.tr

**Keywords:** sitagliptin, radiation, apoptosis, oxidative stress, histopathology

## Abstract

Background: Radiation-induced tissue degeneration is the most important side effect of radiotherapy. Sitagliptin with its anti-inflammatory and antioxidant capacity was tested in alleviating the radiation-induced cellular degeneration in kidney and testis tissues. Methods: Wistar albino rats were divided into four groups as control, radiation (RT), radiation + sitagliptin (RT + SGT), and sitagliptin + radiation (SGT + RT). The RT group received 8 Gy radiation. Sitagliptin was applied per os at a 10 mg/kg dose for 14 days to the SGT groups either after or before radiation. Results: Radiation induced marked oxidative stress in kidney and testis tissues, whereas sitagliptin partially restored several antioxidant parameters in the kidney and reduced MDA levels in the testis. Histologically, radiation caused degenerative changes in the renal tubules and glomerulus and the testicular seminiferous tubules, while sitagliptin treatment attenuated these changes in both organs. Caspase-3 expression increased significantly after radiation treatment in the kidney without substantial improvement by sitagliptin; however, VEGF expression, which was markedly reduced by radiation in both tissues, was restored in sitagliptin-treated groups. FGF expression suppressed in all irradiated groups as compared to the control with no significant differences among them. Conclusions: Overall, the results indicated that sitagliptin can be used to attenuate the degenerative effects induced by radiation. Sitagliptin use after radiation as compared to the before use showed significantly better results especially in the kidney tissue.

## 1. Introduction

Radiation-induced tissue degeneration may occur as a consequence of radiotherapy and accidental or incidental exposure to ionizing radiation [1]. Most commonly, as a result of radiotherapy for cancer treatment and allogeneic hematopoietic stem cell transplantation purposes, patients may encounter local or total body irradiation. Although the overwhelming improvements in chemotherapy for cancer treatment, radiotherapy is still an unavoidable method to be used since it does not rely on the blood supply of the site, and the procedure is not affected by obstacles such as absorption, metabolism, and clearance of the drug [2]. Radiation therapy benefits from the ability of radiation beams to kill cancer cells. Despite its widespread use in malignant tumors, radiotherapy, due to its non-selective effect, inevitably causes the destruction of healthy cells in close proximity to the cancer cells [3]. This is the most significant limitation of radiotherapy and, therefore, requires selective focusing along with suitable dose regimens. Despite advances in imaging methods and achievements in more selective focusing, radiation-induced cell damage can still take place to some degree. Since the discovery of X-rays, the potentially harmful effects of these beams have been extensively investigated. Studies indicate that ionizing radiation can have direct and indirect damaging effects on tissues. It is now clear that radiation-induced tissue degeneration is a complex process, and DNA damage, oxidative stress, induction of various inflammatory signals, and cell death via necrosis, apoptosis, necroptosis [4], pyroptosis [5], and ferroptosis [6] may play a role. The occurrence of DNA double-strand break is the most significant DNA damage, followed by base damage and cross-linking [7,8]. Radiation may also ionize the water molecules in cellular compartments, yielding reactive oxygen species that may act on DNA, proteins, and lipids in cellular membranes and destroy their morphology and functions [9]. This intricate complexity of radiation-induced cellular degeneration makes it difficult to target a simple and straightforward solution to overcome the evident side effects of radiotherapy. Various approaches to minimize or limit radiation-induced tissue damage are still being widely investigated [10,11]. It has been suggested that sitagliptin, an FDA-approved oral hypoglycemic agent, may have a beneficial effect on radiation-induced cellular damage [12]. Sitagliptin is a selective dipeptidyl peptidase 4 (DPP4) inhibitor, and it inhibits the degradation of the endogenous incretin hormones glucagon-like peptide-1 (GLP-1) and glucose-dependent insulinotropic polypeptide (GIP), hence, promoting insulin secretion while inhibiting glucagon secretion [13]. It has been shown that radiation increases DPP4 activity in bone marrow cells in mice, and inhibition or knocking out of DPP4 before irradiation enhances hematopoiesis by preventing cellular damage [14]. Sitagliptin also alleviated radiation-induced intestinal damage [15] and hematopoietic injury in mice [12]. Although the anti-inflammatory and antioxidant effects of sitagliptin were indicated, the search for action on radiation-induced cellular damage is still under investigation. In this study, we aimed to investigate the effect of sitagliptin on radiation-induced cellular damage in the testis and renal tissues.

## 2. Materials and Methods

### 2.1. Animals and Experimental Protocol

Ethical approval of the study was provided by the Institutional Animal Ethics Committee of Inonu University (Approval code: 2021/6-4; Approval date: 11 March 2021). In this study, a total of 38 adult male Wistar albino rats weighing 200–250 g, obtained from the Laboratory Animal Production and Research Center of Inonu University, were divided into four groups: control, radiation (RT), radiation + sitagliptin (RT + SGT), and sitagliptin + radiation (SGT + RT). The control group included 8 rats, while the other groups comprised 10 rats each. All animals were housed in a temperature (23 ± 2 °C) and humidity (55 ± 5%) controlled environment throughout the experiment. Standard rat diet (ARDEN, Ankara, Türkiye) and water were provided *ad libitum*. The control group was given 1 mL of distilled water as the vehicle diluent for 14 days. Rats in the RT group were irradiated with a single dose of 8 Gy using 6 MV X-rays delivered by a linear accelerator (Varian DHX). The irradiation was performed with the SAD technique, targeting a depth of 3 cm with a μ value of 741. Radiotherapy was administered as whole-pelvis irradiation covering both the kidneys and testes. Rats in the RT group were also given 1 mL of distilled water, as in the control group, for 14 days. The RT + SGT group had a single dose of 8 Gy radiation and then 10 mg/kg oral sitagliptin [16] for 14 days. SGT + RT group received a single dose of 8 Gy radiation following 10 mg/kg oral sitagliptin for 14 days.

### 2.2. Biochemical Analyses

At the end of the experimental periods, all rats were euthanized under intraperitoneal injection of 75 mg/kg ketamine and 8 mg/kg xylazine anesthesia. Intracardiac blood was collected, and serum was obtained. Kidney and testis samples were collected and freshly homogenized, and the homogenates were centrifuged to obtain clear supernatants. Tissue levels of malondialdehyde (MDA), glutathione (GSH), and superoxide dismutase (SOD), catalase (CAT), glutathione peroxidase (GPx), total antioxidant status (TAS), total oxidant status (TOS), and oxidative state index (OSI) were determined. MDA level in tissues was detected according to the method described by Mihara and Uchiyama [17], and the results were given as nanomoles per gram of wet tissue (nmol/gwt). GSH level was determined following the method of Ellman [18], in which reduced GSH was reacted with 5,5-dithiobis-2-nitrobenzoic acid, and the level of it was measured spectrophotometrically at 410 nm wavelength, and the results were given as nmol/gwt. SOD activity in tissue samples was detected by the method mentioned by Sun et al. [19] and the results were given as U/g protein. CAT activity was measured based on the method described by Aebi [20] and Lück [21] in that the enzyme activity was determined by the decomposition of 30 mmol/L H_2_O_2_ in 50 mmol/L potassium phosphate buffer (pH 7.0) at 240 nm at 15 s intervals for 150 s by a spectrophotometer. The difference in absorbance per unit time is accepted as the measurement of the CAT activity. The results were given as the rate constant (k) of a first-order reaction per gram of protein in tissues (K/g protein). GPx enzyme activity was determined spectrophotometrically at 340 nm according to the Paglia and Valentine method [22] and results were given as U/mg protein. TOS (Catalog number: MK221460) and TAS (Catalog number: MS22128A) were determined by a colorimetric method according to the instructions provided by the Rel Assay Diagnostic Company. OSI was calculated by dividing the TOS value by the TAS value.

### 2.3. Histopathology

Kidney and testis samples collected for histopathological examination were fixed in 10% formaldehyde and then embedded in paraffin. With the help of a microtome, 5 µm sections were cut from the paraffin blocks. The tissue sections were routinely stained with hematoxylin and eosin. All sections were assessed by a single observer blinded using a light microscope (Leica DFC280, Wetzlar, Germany). In histological evaluation, for each tissue section, 10 different fields were viewed under a 40× objective and photographed, and the images were analyzed with the help of Leica Q image analysis system (Leica Micros Imaging Solution Ltd., Cambridge, UK).

Histopathology scores were determined for both kidney and testis tissues for each group. Renal injury was assessed by the criteria of intertubular hemorrhage, peritubular infiltration, glomerular capillary collapse, and degeneration of tubule cells. Scoring of each parameter was as 0: none, 1: mild, 2: moderate, and 3: severe. The results were presented as median (min–max). The Johnsen [23] testicular histologic injury score method was used to assess the functional state of the testicular tissues. The diameter of the seminiferous tubules and the thickness of the germinal epithelium layer were also measured and compared among the groups [24].

### 2.4. Immunohistochemical Analyses

Vascular endothelial growth factor (VEGF) and fibroblast growth factor (FGF) immunoreactivity were investigated in kidney and testis tissue sections. Caspase-3 reactivity was also studied in kidney tissue samples. For immunohistochemical analyses, the tissue sections were routinely deparaffinized and then rehydrated. Antigen retrieval was provided by 0.01 M citrate solution (pH 6.0) and boiling the sections for 15–20 min. Endogenous peroxidase activity was blocked by 3% H_2_O_2_ treatment at room temperature for 10 min. The sections were routinely rinsed with phosphate-buffered solution (PBS), and then ultra V block was applied for 5 min to block non-specific antibody binding. Then, tissue sections were incubated with primary antibodies to VEGF (diluted 1:100, Santa Cruz, Catalog no: sc-7269), FGF-2 (diluted 1:100, Santa Cruz, Catalog no: sc-365106), and caspase-3 (diluted 1:100, Santa Cruz, Catalog no: sc-56053) at 37 °C for 1 h. Following rinses with PBS, biotinylated secondary antibody and then streptavidin peroxidase solution (Thermo Fisher Scientific, Fremont, CA, USA) were applied each at 37 °C for 10 min. Diaminobenzidine was used as the chromogen (Thermo Fisher Scientific, Fremont, CA, USA) at 37 °C for 2–8 min to visualize the immunoreactivity. Finally, the sections were stained with hematoxylin, covered, and observed under a light microscope. Staining distribution was assessed semiquantitatively as 0: 0–25%, 1: 26–50%, 2: 51–75%, and 3: 76–100%. The staining intensity was determined as 0: none, 1: weak, 2: moderate, and 3: severe. Immunoreactivity scores were calculated by multiplying the staining distribution by the staining intensity.

### 2.5. Statistical Analyses

Based on data from the literature [25], to detect a statistically significant difference between the groups for biochemical variables, the Type I error rate was set at 0.05, the Type II error rate at 0.20 (80% power), the effect size was 1.01 (interpreted as large), and a two-tailed alternative hypothesis was applied. Sample size calculations indicated that at least 8 rats were required per group. To account for potential mortality during radiotherapy, 2 additional rats were included in the groups receiving RT. IBM SPSS Statistics Version 25 computer software was used for the statistical analysis. The normality of the data distribution was assessed using the Kolmogorov–Smirnov test. For data with a normal distribution, intergroup differences were analyzed using one-way analysis of variance (ANOVA), and multiple comparisons were performed using the Tukey HSD or Tamhane test, depending on the homogeneity of variances. For data with a non-normal distribution, differences were analyzed using the Kruskal–Wallis H test, and pairwise comparisons were performed using the Mann–Whitney U test. Data with a normal distribution were presented as mean ± standard deviation, while data with a non-normal distribution were presented as median (minimum–maximum) (Median (Min–Max)). *p* < 0.05 was considered statistically significant.

## 3. Results

### 3.1. Biochemical Analyses

The results of biochemical analyses are given in Table 1 and Table 2 for the kidney and testis, respectively. In kidney tissues, MDA, GSH, SOD, CAT, GPx, TAS, TOS, and OSI levels in the RT group significantly differed from those of the control group (*p* < 0.05). As compared to the RT group, levels of GSH, SOD, CAT, TAS, TOS, and OSI were significantly different in the RT + SGT group, while only levels of GSH, TAS, TOS, and OSI were significantly different in the SGT + RT group (*p* < 0.05). There was no significant difference between RT + SGT and SGT + RT groups at all the parameters investigated (*p* > 0.05).

In testis tissues, MDA, SOD, CAT, GPx, TOS, and OSI levels were significantly different between the control and RT group (*p* < 0.05). MDA levels both in the RT + SGT group and SGT + RT group were significantly lower than those of the RT group (*p* < 0.05). Only levels of CAT and TOS in the RT + SGT group were significantly different from those of the RT group (*p* < 0.05). Except for the level of MDA, there was no significant difference between RT + SGT and SGT + RT groups at all the parameters investigated (*p* > 0.05). GSH levels were not different in all the groups (*p* > 0.05)

### 3.2. Histopathology

**Figure 1:**
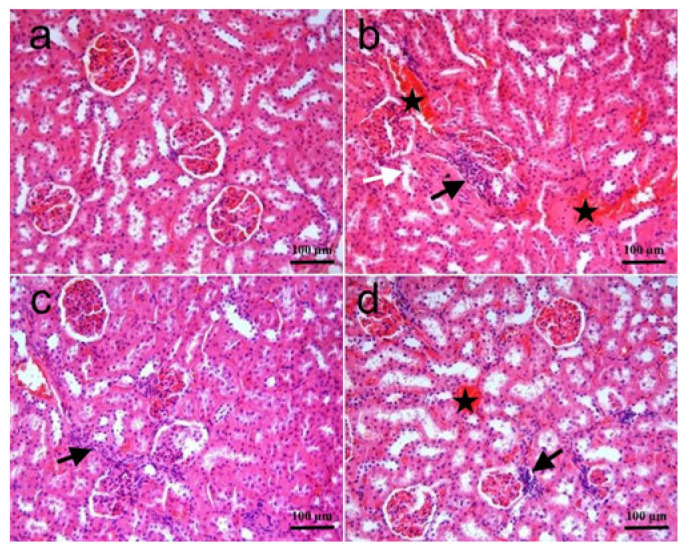
Kidney. (**a**) **Control group:** Normal histomorphology; (**b**) **RT group:** Glomerular collapse, severe hemorrhages (star), and inflammatory cellular infiltration (black arrow) in the interstitium and some degenerate tubules (white arrow)l; (**c**) **RT + SGT group:** Inflammatory cellular infiltration (black arrow). Note no hemorrhage and mild tubular and glomerular changes; (**d**) **SGT + RT group:** Foci of interstitial hemorrhages (star), inflammatory cellular infiltration (black arrow) and mild tubular degeneration. H & E.

The histopathological changes in kidney tissues were shown in Figure 1a–d, and the differences among the groups were summarized in Table 3. Kidney sections showed normal histomorphology in the control group. In the RT group, hemorrhage and cellular infiltrations in cortical intertubular areas, collapse in some glomeruli, and degeneration in some tubule epithelia were noted. These histopathological changes were significantly higher in this group as compared to the control. In the RT + SGT group, some degree of histopathological changes was observed in glomeruli and tubule epithelia. Small areas of hemorrhage and some cellular infiltrations were also noted. The degree of these changes was significantly lower than that of the RT group (*p* < 0.05). In the SGT + RT group, the degree of the histopathological changes was mostly similar to that of the RT + SGT group. Histopathological changes in this group were significantly lower than those of the RT group (*p* < 0.001). In terms of histopathological scores, there was no significant difference between the RT + SGT group and the SGT + RT group (*p* > 0.05).

**Figure 2:**
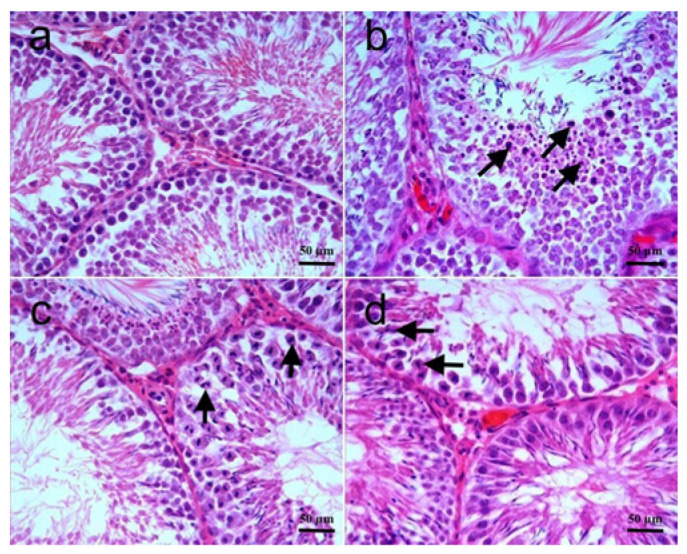
Testis. (**a**) **Control group:** Normal histomorphology; (**b**) **RT group:** Note that many cells of the spermatogenic series (arrows) stopped at different developmental stages, detached prematurely, and accumulated in the lumen of some seminiferous tubules. The number of mature spermatocytes is clearly less; (**c**) **RT + SGT group:** Degenerate cells (arrows) showing bizarre shapes trapped in different stages of meiosis; (**d**) **(SGT + RT group):** Similarly to the RT + SGT group, showing numerous degenerated cells (arrows). H & E.

The histopathological changes in testis tissues are shown in Figure 2a–d, and the differences among the groups are summarized in Table 4. Testis tissue samples of the control group showed normal histomorphology. In the RT group, the histomorphology of some seminiferous tubules was significantly distorted and lost their round to ovoid conformation, and there were large gaps between the tubules, enlarging the interstitial space. In some seminiferous tubules, the number of cells forming the spermatogenetic series decreased and showed irregular placement. In most tubules, varying numbers of degenerated cells characterized by eosinophilic cytoplasm were noted. Cells of the spermatogenic series at different developmental stages were seen to detach prematurely and accumulate in the lumen of some tubules. Histomorphological changes were significantly decreased both in RT + SGT and SGT + RT groups as compared to the RT group (*p* < 0.0001). In both groups, degenerate cells trapped in different stages of meiosis, having bizarre shapes, were noted. Similar histopathological changes were observed in both RT + SGT and SGT + RT groups, and there was no significant difference between the two groups (*p* > 0.05).

### 3.3. Immunohistochemical Analyses

#### 3.3.1. Kidney Tissue

**Figure 3:**
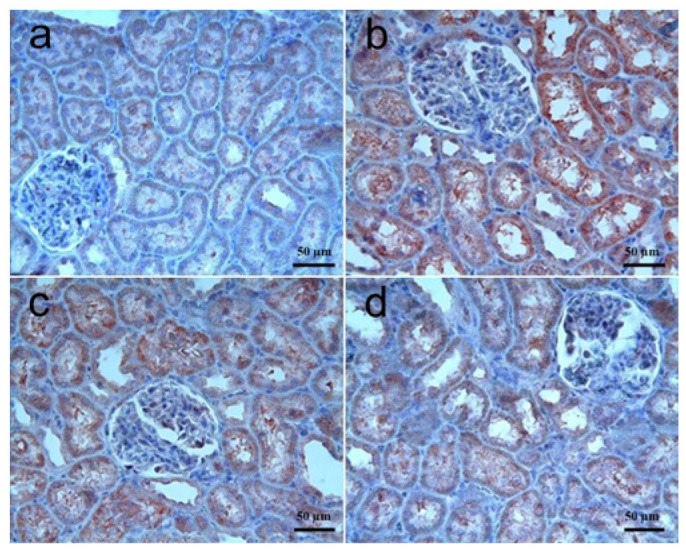
Kidney, caspase-3. (**a**) **Control group:** No immunoreactivity; (**b**) **RT group:** Strong caspase-3 immunoreactivity in almost all renal tubule epithelia; (**c**) **RT + SGT group:** Moderate-to-strong caspase-3 immunoreactivity in renal tubules; (**d**) **SGT + RT group:** Moderate-to-strong caspase-3 immunoreactivity in renal tubules. IHC.

Caspase-3: The results of immunohistochemical staining for caspase-3 in kidney tissues are shown in Figure 3a–d, and the statistical significances were summarized in Table 3. There was no caspase-3 activity in the control group. In the RT group, strong caspase-3 immunoreactivity was observed in the tubule epithelia of some cases, and the staining score was significantly higher in the RT group as compared to the control group (*p* < 0.0001). Caspase-3 immunoreactivity in RT + SGT and SGT + RT groups was generally similar to that of the RT group in that moderate-to-strong immunoreactivity was observed, and there was no significant difference among these three groups in terms of the severity and the pattern of the immunoreactivity in kidney tissues (*p* > 0.05).

**Figure 4:**
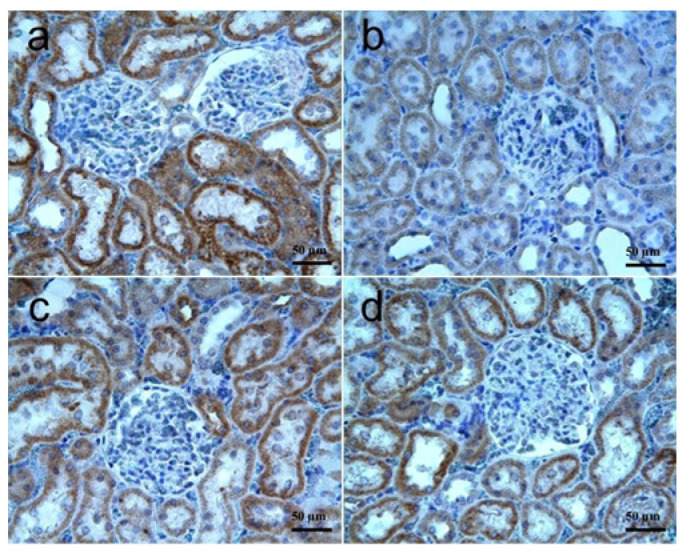
Kidney, VEGF; (**a**) **Control group:** Strong immunoreactivity most prominently in renal tubule epithelia; (**b**) **RT group:** Significantly decreased VEGF immunoreactivity as compared to the control group, yet staining distribution was similar; (**c**) **RT + SGT group:** Strong immunoreactivity similar to the control group; (**d**) **SGT + RT group:** Strong immunoreactivity similar to the control group. IHC.

VEGF: The results of immunohistochemical staining for VEGF in kidney tissues are shown in Figure 4a–d, and the statistical significances are summarized in Table 3. VEGF immunoreactivity was detected in the cytoplasm of the cortical tubular epithelia in all groups. There was no detectable immunoreactivity in glomeruli and the interstitium. VEGF immunoreactivity score was significantly lower in the RT group as compared to the other three groups (*p* < 0.0001). Although tubular epithelia all showed similar staining distribution, the staining intensity was significantly lower in the RT group. No significant difference among the control, RT + SGT, and SGT + RT groups was detected for VEGF expression (*p* > 0.05).

**Figure 5:**
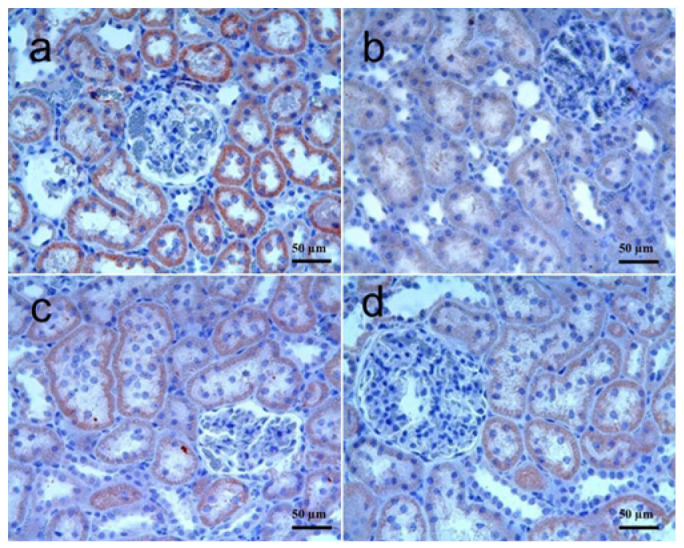
Kidney, FGF; (**a**) **Control group:** Strong immunoreactivity in renal tubule epithelia; (**b**) **RT group:** Significantly decreased FGF immunoreactivity as compared to the control group; (**c**) **RT + SGT group:** Weak immunoreactivity similar to the RT group; (**d**) **SGT + RT group:** Weak immunoreactivity similar to RT and RT + SGT group. IHC.

FGF: The results of immunohistochemical staining for FGF in kidney tissues are shown in Figure 5a–d, and the statistical significances are summarized in Table 3. FGF immunoreactivity was observed cytoplasmically in cortical tubular epithelia in all groups. Strong FGF immunoreactivity was detected in the control group. Immunoreactivity scores of the RT, RT + SGT, and SGT + RT groups were lower than those of the control group (*p* < 0.0001), and there were no significant differences among the three groups with radiation exposure (*p* > 0.05).

#### 3.3.2. Testis Tissue

**Figure 6:**
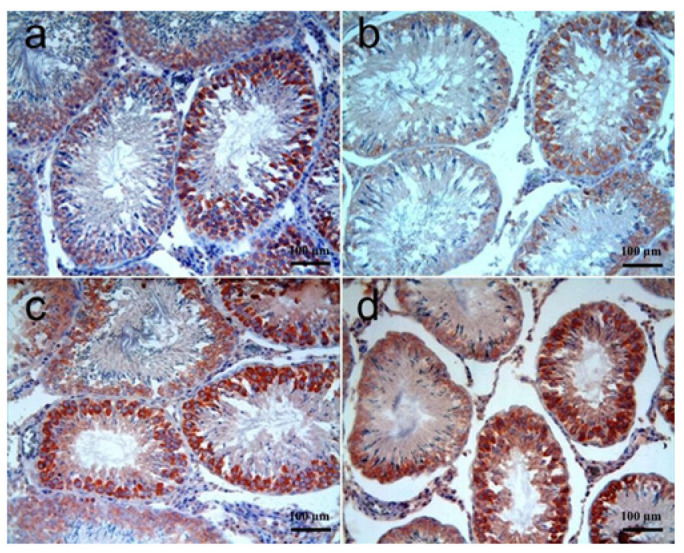
Testis, VEGF; (**a**) Control group: Strong immunoreactivity in primary spermatocytes in seminiferous tubules and weak-to-moderate immunoreactivity in Leydig cells; (**b**) **RT group:** Weak immunoreactivity both in spermatogenetic cells in seminiferous tubules and Leydig cells; (**c**) **RT + SGT group:** Strong immunoreactivity pattern similar to the control group; (**d**) **SGT + RT group:** Strong immunoreactivity similar to the control group. IHC.

VEGF: The results of immunohistochemical staining for VEGF in testis tissues are shown in Figure 6a–d, and the statistical significances are summarized in Table 4. In the control group, strong immunoreactivity was observed in primary spermatocytes in seminiferous tubules. Weak-to-moderate immunoreactivity was also noted in Leydig cells. In the RT group, parallel to the distorted histomorphology, VEGF immunoreactivity was significantly decreased compared to the control (*p* < 0.0001) and only limited to the basal spermatocytes. VEGF immunoreactivity score of the testis tissues of RT + SGT and SGT + RT groups were detected to be comparably higher than that of the RT group (*p* < 0.0001).

**Figure 7:**
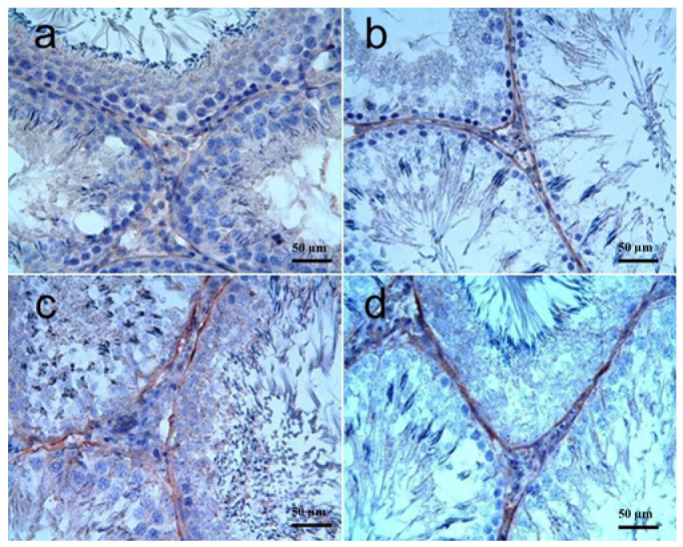
Testis, FGF; (**a**) Control group: Moderate-to-strong immunoreactivity in peritubular cells and weak immunoreactivity in some spermatocytes; (**b**) **RT group:** Significantly decreased immunoreactivity in peritubular cells and no reactivity in spermatocytes; (**c**) **RT + SGT group:** Weak immunoreactivity similar to RT group; (**d**) **SGT + RT group:** Weak immunoreactivity similar to RT and RT + SGT groups. IHC.

FGF: The results of immunohistochemical staining for FGF in testis tissues are shown in Figure 7a–d, and the statistical significances are summarized in Table 4. FGF immunoreactivity was mainly observed in peritubular cells in all the groups. Some spermatocytes also showed weak FGF immunoreactivity. In the RT group, FGF expression was significantly decreased as compared to the other groups (*p* < 0.0001). FGF immunoreactivity in RT + SGT and SGT + RT groups was similar to each other, and no significant difference was noted between them (*p* > 0.05).

## 4. Discussion

In this study, sitagliptin was shown to have protective effects on radiation-induced cellular degeneration in renal and testicular tissues of rats. Radiation is known to cause prominent cellular degeneration in almost all types of cells. Most strikingly, cells with higher mitotic capability are more sensitive to radiation-induced cellular degeneration [26]. Hence, spermatocytes, which have to go through both meiotic and mitotic divisions, and kidney tubule epithelia, which possess high regenerative capability, are highly sensitive to radiation [27]. Kidney tubule epithelia and spermatocytes also have high metabolic activity and are, hence, prone to cellular degeneration caused by numerous insults [28]. In this investigation, we have also observed severe degenerative changes as a result of the radioactive beam in both tissues.

Apoptotic cell death is commonly reported in radiation-induced tissue degeneration [29,30]. Increased caspase-3 immunoreactivity in our study proves the incidence of apoptosis in kidney tissue samples as a result of radiation. Apoptotic cells were also detected in radiation plus sitagliptin-treated groups, and there was no statistical difference in apoptotic cell death index as compared to the only radiation given group. This result indicates that in kidney tissue, sitagliptin application does not provide enough protection for cells from apoptosis. Apoptotic cell death can be classically activated through DNA damage and inactivation of the G2/M cell cycle checkpoint [31]. The expression of Fas is also upregulated by irradiation [32]. Depending on the dose, cell death can take place via necrosis [33] or apoptosis [8]. Other cell death types can also be involved in tissue degeneration as a result of radiation [34]. In histopathological view, significant cellular degeneration characterized by glomerular collapse, tubular degeneration, and inflammatory cellular infiltrations indicates that at the given radiation dose, significant tissue degeneration takes place, though cellular death is yet limited. Inflammatory cellular infiltration and following cytokine-mediated tissue degeneration are often described in radiation-induced tissue degeneration, and, hence, our results comply with the previous studies [8]. In spite of the presence of inflammatory cellular infiltration, the high expression level and distribution of caspase-3 immunoreactivity in all experimental groups indicate that, in the experimental setup, the applied radiation dose induces strong cellular degeneration, as sitagliptin application does not provide the expected protective action. However, the results of VEGF and FGF immunoreactivities may suggest that at lower doses of radiation, sitagliptin might cause a prominent reduction in apoptotic cell death.

Several trophic factors play important roles in the survival, development, and homeostasis of the cells. Depending on the type and function of a given cell, the need for the growth factors shows changes. VEGF is an endothelial growth factor and plays crucial roles in endothelial cell proliferation, differentiation, and survival. It also mediates vasodilation and increases endothelial permeability [35]. Its significant involvement in cancer growth and metastasis was well known due to its involvement in microvasculature development [36,37]. FGF, another important growth factor that is expressed widely due to its essential roles in matrix remodeling and the expression of several other cytokines from fibroblasts and other cells [38]. VEGF in renal tissues is mainly expressed in the tubule epithelia and less frequently in podocytes [39], while FGF-2 is expressed in tubular cells, glomerular parietal epithelial cells, and arterial endothelial cells of the kidney [40]. Decreased VEGF expression was reported as a result of various degenerative insults [41]. We have detected significant decreases in both VEGF and FGF expressions in renal tubules. These tubules are known to be highly prone to several injuries, including radiation [42]. Increased caspase-3 immunoreactivity and changes in renal histomorphology in this study support the concept of susceptibility of the renal tissue to injury.

Radiotherapy is well known to cause fertility problems both in males and females. Radiation-induced early side effects can be extremely dangerous as they cause significant cellular degeneration and death at high doses [43]. Similar to the renal tubular epithelia, seminiferous tubules in testis tissue were reported to be highly sensitive to radiation [27]. Severe degenerative changes characterized by distorted and lost spermatogenetic cells were also detected in irradiated rats in this study. Leydig cells were reported to be more resistant to radiation, but at high doses, degenerative changes in these cells were associated with decreased testosterone production, and hence with erectile dysfunction, reduced stamina, and testicular infertility [44,45,46]. Distorted testicular morphology with widened interstitial space in irradiated rats is most probably due to the destruction of Leydig cells in this study.

Early side effects of radiation at lower doses can be reversible in tissues with a high turnover rate. Hence, substances that have anti-inflammatory and antioxidant potentials have a good chance of preventing cellular degeneration [47]. Although sitagliptin was reported to have some degree of effectiveness in radiation-induced testicular degeneration [12], we have detected some limited protection, most probably due to the development of fast and severe tissue degeneration that could not be reversed or prevented by the action of the anti-inflammatory capacity of sitagliptin.

Radiation-induced cellular degeneration in endothelial cells may also impact other cells [8,48]. Due to their eminent function for supplying blood to the tissues as well as important trophic factors for other cells, degenerative changes in endothelial cells might play a significant role in overall tissue homeostasis. We have detected significant decreases in the expressions of VEGF and FGF in both renal and testicular tissues of the irradiated rats as compared to the control group. In both organs, expressions of these growth factors showed significant increases in sitagliptin-treated groups both before and after the radiation application, and the expression levels in both groups were similar to those of the control group. These findings indicate that sitagliptin significantly improved the expression of VEGF and FGF in the irradiated rats.

DPP4 is a glycoprotein expressed on the surface of most cells and plays important roles in immune regulation, signal transduction, and cell death via apoptosis [49]. DPP4 cleaves substrates such as growth factors, chemokines, neuropeptides, and vasoactive peptides; hence, it mostly inhibits their actions [50,51]. It has both catalytic and non-catalytic activities. Inhibition of its action was, therefore, associated with several cellular processes. DPP4 inhibitors are commonly used in diabetes treatment, but their effects on other conditions, such as cardiovascular disorders and inflammatory diseases, are also under investigation [52]. Sitagliptin, as a DPP4 inhibitor, was also suggested to be used in the prevention of radiation-induced cellular degeneration [12]. Sitagliptin’s action on several insults was associated with its anti-inflammatory and antioxidant potential [53,54]. Cytokine secretion and its complex interactions with the key inflammatory and parenchymal cells are at the core of this preventive action [52]. Linagliptin, another DPP4 inhibitor, was suggested to have a cardioprotective role through FGF-2/EGR-1 pathway in mice with dietary obesity, and capillary rarefaction was suggested to be important in this process [55]. FGF-2 was indicated to significantly attenuate apoptosis of the kidney tissue after ischemia/reperfusion injury [56]. FGF-2 was also indicated to be produced by renal tubular cells to act as a paracrine factor after cisplatin-induced nephrotoxicity, showing the importance of this growth factor in cellular survival and repair [57]. Decreased FGF level both in the kidney and testis tissues of the irradiated rats was altered with sitagliptin application in the current study. This result suggests that the cytoprotective potential of sitagliptin may occur through increased expression of VEGF and FGF.

Oxidative stress is the most common event in the process of cellular degeneration as a result of numerous insults [58]. Radiation-induced oxidative stress develops due to altered production of cellular proteins that are involved in cellular defense, as well as ionization of water molecules that causes the excess production of reactive oxygen and nitrogen species [1]. Radiation-induced tissue degeneration was indicated to significantly elevate both intracellular and extracellular levels of reactive oxygen species [59,60]. These reactive species may then interact with important cellular structures, including membranes, proteins, and the genetic material, causing both functional and morphological changes, which may end up with the death of the cell [61]. In the current study, we also observed prominent cellular degeneration and death in both kidney and testis tissues, assessed by histopathological changes as well as biochemical markers.

Oxidative stress markers are commonly used to assess the status of tissues to the degenerative insults. MDA as an indicator of lipid peroxidation via reactive oxygen species is the most commonly used marker for cellular degeneration [62]. MDA level both in the kidney and testis tissues of irradiated rats increased significantly as compared to the control group, indicating severe degenerative changes. Sitagliptin application was detected to be effective in reducing the MDA level in testis tissue, but not in kidney tissue. However, other biochemical markers significantly improved in the kidney tissue of the sitagliptin given group after radiation induction. On the other hand, in the testis tissue of the sitagliptin-treated rats, these markers did not show any improvements as compared to the rats treated with radiation only. Supported by the findings of the histopathological observations, sitagliptin was more effective in the kidney tissue in this study. High sensitivity of the testis tissue to degenerative insults and the need for more time to repair most probably did not lead to attenuation of the cellular changes in this organ. The less protective effect of sitagliptin in the testis tissue compared to the renal tissue is that DPP4 is highly expressed in the kidney and, therefore, causes significant inhibition in the renal tissue, while in the testis tissue, it is less expressed [63]. Another possible explanation for the less protective effect in the testis tissue can be explained by the presence of the blood–testis barrier, which may limit the penetration of the drug to the tissue, not allowing it to act on it.

## 5. Conclusions

In conclusion, sitagliptin shows some protective effect in radiation-induced cellular degeneration in the kidney and testis tissues. Its protective potential was observed to be better in kidney tissue as compared to the testis and it was more effective if used after radiation application. It was observed that oxidative stress-mediated cellular degeneration and death via apoptosis play significant roles in radiation-induced tissue damage, and sitagliptin, with its anti-inflammatory and antioxidant potential, can attenuate the cellular damage in kidney tissues, but less so in testicular tissue.

## Figures and Tables

**Table 1:** Kidney tissue biochemical parameters for oxidative stress and the statistical comparisons among the groups.

	Parameters							
Groups	MDA (nmol/gwt) (Mean ± SD)	GSH (µmol/gwt) (Mean ± SD)	SOD (U/mg Protein) (Mean ± SD)	CAT (K/g Protein) (Mean ± SD)	GPx (U/mg Protein) (Mean ± SD)	TAS (mmol Trolox Equiv./L) (Median (Min–Max))	TOS (µmol H_2_O_2_ Equiv./L) (Mean ± SD)	OSI (AU: Arbitrary Unit) (Mean ± SD)
C	26.53 ± 2.9	18.55 ± 1.5	5.98 ± 1.5	154.72 ± 22.0	1.14 ± 0.3	2.95 (2.8–3.1)	9.69 ± 1.0	3.29 ± 0.4
RT	50.56 ± 15.1	10.90 ± 2.3	3.19 ± 0.3	70.80 ± 29.2	0.48 ± 0.2	1.79 (1.2–2.2)	14.96 ± 2.2	8.80 ± 2.7
RT + SGT	33.99 ± 10.5	14.28 ± 2.7	4.21 ± 0.5	108.75 ± 25.5	0.75 ± 0.2	2.72 (1.8–2.9)	11.39 ± 2.2	4.35 ± 0.7
SGT + RT	40.72 ± 7.3	15.57 ± 2.2	3.92 ± 0.9	93.97 ± 30.8	0.75 ± 0.2	2.42 (2.2–2.8)	9.70 ± 1.6	4.00 ± 0.7
	Statistical Comparisons (*p* value)							
C vs. RT	0.015 *	0 *	0.007 *	0 *	0 *	0.001 *	0 *	0.004 *
C vs. RT + SGT	0.430	0.004 *	0.079	0.01 *	0.005 *	0.005 *	0.271	0.012 *
C vs. SGT + RT	0.004 *	0.057	0.043 *	0.001 *	0.005 *	0.001 *	1	0.155
RT vs. RT + SGT	0.141	0.025 *	0.002 *	0.042 *	0.083	0.003 *	0.003 *	0.013 *
RT vs. SGT + RT	0.560	0.001 *	0.335	0.336	0.079	0.002 *	0 *	0.008 *
RT + SGT vs. SGT + RT	0.654	0.658	0.968	0.697	1	0.103	0.276	0.906

* Statistically significant (*p* < 0.05). MDA: malondialdehyde; GSH: glutathione; SOD: superoxide dismutase; CAT: catalase; GPx: glutathione peroxidase; C: control; RT: radiation; SGT: sitagliptin; TAS: total antioxidant status; TOS: total oxidant status; OSI: oxidative stress index; gwt: gram wet tissue.

**Table 2:** Testis tissue biochemical parameters for oxidative stress and the statistical comparisons among the groups.

Parameters								
Groups	MDA (nmol/gwt) (Median (Min–Max))	GSH (µmol/gwt) (Mean ± SD)	SOD (U/mg Protein) (Mean ± SD)	CAT (K/g Protein) (Mean ± SD)	GPx (U/mg Protein) (Mean ± SD)	TAS (mmol Trolox Equiv./L) (Median (Min–Max))	TOS (µmol H_2_O_2_ Equiv./L) (Mean ± SD)	OSI (AU: Arbitrary Unit) (Mean ± SD)
C	18.25 (15.6–21.8)	16.35 ± 2.4	7.29 ± 1.9	175.18 ± 24.4	0.61 ± 0.2	2.32 (2.1–2.7)	9.90 ± 1.9	4.28 ± 1.0
RT	31.94 (22.1–34.7)	13.90 ± 2.1	4.65 ± 0.5	93.04 ± 35.6	0.32 ± 0.1	2.29 (1.4–2.5)	14.01 ± 1.4	6.67 ± 1.4
RT + SGT	18.86 (15.6–23.9)	15.24 ± 2.5	5.88 ± 1.5	154.43 ± 45.9	0.45 ± 0.1	2.39 (1.6–2.6)	10.93 ± 2.4	5.04 ± 1.5
SGT + RT	21.98 (18.0–23.2)	14.23 ±1.4	6.69 ± 2.2	114.33 ± 28.4	0.51 ± 0.2	2.51 (1.3–2.8)	12.12 ± 1.4	5.37 ± 1.7
	Statistical comparisons (*p* value)							
C vs. RT	0.001 *	0.124	0.031 *	0 *	0.01 *	0.344	0.001 *	0.012 *
C vs. RT + SGT	0.529	0.727	0.529	0.631	0.283	1	0.668	0.717
C vs. SGT + RT	0.006 *	0.219	0.993	0.008 *	0.683	0.226	0.091	0.443
RT vs. RT + SGT	0.001 *	0.601	0.262	0.007 *	0.404	0.207	0.011 *	0.128
RT vs. SGT + RT	0.002 *	0.989	0.194	0.612	0.124	0.052	0.183	0.286
RT + SGT vs. SGT + RT	0.027 *	0.784	0.957	0.117	0.893	0.207	0.568	0.968

* Statistically significant (*p* < 0.05). MDA: malondialdehyde; GSH: glutathione; SOD: superoxide dismutase; CAT: catalase; GPx: glutathione peroxidase; C: control; RT: radiation; SGT: sitagliptin; TAS: total antioxidant status; TOS: total oxidant status; OSI: oxidative stress index; gwt: gram wet tissue.

**Table 3:** Kidney tissue histopathological and immunohistochemical scores (Median (Min–Max)).

Parameters	Groups			
	C	RT	RT + SGT	SGT + RT
Hemorrhage	0 (0–0)	1 (0–3) ^a^	0 (0–2) ^b^	0 (0–2) ^b^
Cellular infiltration	0 (0–0)	1 (0–3) ^a^	0 (0–2) ^b^	0 (0–2) ^b^
Glomerular collapse	0 (0–0)	1 (0–3) ^a^	0 (0–2) ^b^	0 (0–2) ^b^
Tubular cellular degeneration	0 (0–0)	1 (0–2) ^a^	0 (0–2) ^b^	0 (0–2) ^b^
Caspase-3 immunoreactivity	0 (0–0)	1 (0–2) ^a^	1 (0–2) ^a^	1 (0–2) ^a^
VEGF immunoreactivity	1 (0–2)	0 (0–1) ^c^	1 (0–2) ^d^	1 (0–2) ^d^
FGF immunoreactivity	1 (0–2)	0 (0–1) ^c^	0 (0–1)	0 (0–1)

In each row, ^a^ statistically significant difference with control (*p* < 0.0001); ^b^ statistically significant difference with RT group (*p* < 0.0001); ^c^ statistically significant difference with control group (*p* < 0.0001); ^d^ statistically significant difference with RT group (*p* < 0.0001). C: control, RT: radiation, SGT: sitagliptin.

**Table 4:** Testis tissue histopathological and immunohistochemical scores (Median (Min–Max)).

Parameters	Groups			
	C	RT	RT + SGT	SGT + RT
Johnsen score	9 (8–10)	7 (1–10) ^a^	8 (7–10) ^b^	8 (7–10) ^b^
VEGF immunoreactivity	1 (0–2)	0 (0–1) ^c^	1 (0–2) ^d^	1 (0–2) ^d^
FGF immunoreactivity	1 (0–2)	0 (0–1) ^c^	1 (0–2) ^d^	1 (0–2) ^d^

In each row, ^a^ statistically significant difference with the control group (*p* < 0.0001); ^b^ statistically significant difference with RT group (*p* < 0.0001); ^c^ statistically significant difference with control group (*p* < 0.0001); ^d^ statistically significant difference with RT group (*p* < 0.0001). C: control, RT: tadiation, SGT: sitagliptin.

## Data Availability

The original contributions presented in this study are included in the article. Further inquiries can be directed to the corresponding author(s).

## Abbreviations

The following abbreviations are used in this manuscript:

GLP-1	Glucagon-like peptide-1
GIP	Glucose-dependent insulinotropic polypeptide
RT	Radiation
VEGF	Vascular endothelial growth factor
FGF	Fibroblast growth factor
PBS	Phosphate-buffered solution
DPP4	Dipeptidyl peptidase 4
MDA	Malondialdehyde
GSH	Glutathione
SOD	Superoxide dismutase
CAT	Catalase
GPx	Glutathione peroxidase
TAS	Total antioxidant status
TOS	Total oxidant status
OSI	Oxidative state index
